# The reliability of estimating visual working memory capacity

**DOI:** 10.1038/s41598-019-39044-1

**Published:** 2019-02-04

**Authors:** Mengnuo Dai, Yanju Li, Shuoqiu Gan, Feng Du

**Affiliations:** 1 CAS Key Laboratory of Behavioral Science, Institute of Psychology, Chinese Academy of Sciences, Beijing, 100101 China; 2 Department of Psychology, University of Chinese Academy of Sciences, Beijing, 100049 China

## Abstract

The reliability of estimations of working memory capacity has not been thoroughly examined. The present study examined the test-retest reliability for working memory capacity as estimated in a lateralized change detection task, which is frequently used in studies involving electroencephalography. The test-retest correlations between K values for each set size in the two tests varied from 0.502 to 0.757, with test-retest correlations rising as set size increased. The results indicate that individual visual working memory capacity can be reliably estimated in a change detection task. Furthermore, test-retest reliability was higher when the two tests occurred at the same time of day than at different times of day.

## Introduction

Working memory is a temporary storage system under active attentional control^1^. The capacity of working memory is highly limited. For example, on average, people can only hold 3-4 items in visual working memory^2–4^. Although having a limited capacity, working memory has been shown to play an essential role in cognitive functions, such as fluid intelligence^5^, reasoning^6^, reading comprehension^7,8^, second language proficiency of adult learners^9^, and academic attainment^10^.

However, although research has involved an assumption that different kinds of estimation are all reliable measurements for working memory capacity, this has not been thoroughly examined. Initially, researchers focused on verbal working memory by using paradigms such as verbal recall tests^8^, the classic digit N-back task^11^, and the number counting task^12^. The test-retest reliability of working memory capacity estimation based on these tasks has been examined. For example, a study used verbal recall tasks to test 4~15-year-old children and retested them after 5~10 days. The results showed that the verbal recall tasks had an average test-retest reliability of 0.56 for non-word-syllables, 0.72 for words, 0.81 for digits, and 0.61 for sentences and counting numbers^13^. Another study showed that the test-retest reliability of working memory capacity in a spatial N-back paradigm varies from 0.493 to 0.857, depending on the N and whether accuracy or reaction time of the task is calculated^14^.

Studies on visual working memory (VWM) have relied on the change detection task to quantify VWM capacity^2,3^. Participants usually show a limitation of visual working memory capacity at about 3–4 objects’ worth of information^4^, which has been linked with visual search^15^ and multiple-object tracking performance^16^. A study also revealed that individuals with higher VWM capacity are more efficient at excluding unnecessary items during task performance^17^.

Since researchers use an estimation of individual VWM capacity to account for individual differences in other cognitive functions, it is essential to evaluate the reliability of VWM capacity estimation based on the change detection task. However, only a few studies have examined the reliability of VWM capacity estimation. For instance, Johnson *et al*.^18^ examined the test-retest reliability of VWM capacity estimation with a separation of 1.5 years between the two tests. They found a test-retest correlation of 0.77. However, since their study only sampled 31 schizophrenia patients and 14 healthy participants, the representativeness of the result is questionable. Thus, whether VWM capacity estimation is reliable across tests remains essentially unknown.

Test-retest reliability is critical not only for measuring individual differences but for the reproducibility of psychological findings. For example, a large reproducibility project^19^ examined the reproducibility of 100 experiments published in social and cognitive psychology journals. The results showed that only 38 of 100 experiments were rated to have replicated the original results. Thus, the test-retest reliability of VWM capacity estimation provides a baseline level of the reproducibility of VWM capacity estimation given that the subjects and task are the same in the two tests.

Some researchers have suggested that VWM capacity may not have a satisfactory internal consistency across different set sizes in a single test^20^ because estimation of visual working memory capacity relies heavily on the set size (number of to-be-remembered items) of visual stimuli in the change detection task^2,3^. It has also been suggested that change detection tasks using a single-probe (only the possibly-changed square was tested) and using a whole-display-probe (all squares including the possibly-changed one were tested) yield different reliability^20^. However, studies have shown that there is a high correlation between different types of memory materials in the change detection task^21^ and different working memory tasks^22^. Furthermore, Xu *et al*.^23^ conducted one session of single-probe tests with 78 participants every day for 30 continuous days and the 31^st^ session after a month. The correlation coefficientsbetween adjacent sessions varied from 0.53 to 0.81, and importantly they were positively correlated with their session numbers, indicating an increased test-retest reliability as sessions grew in number. However, the test-retest reliability of VWM capacity estimation in a whole-display-probe task has not yet been investigated.

In addition to set size and repeated practice, other factors might also influence the test-retest reliability of VWM measures. For example, it is known that time of day affects people’s ability to retrieve information from long-term memory^24^. A recent study showed that there are also circadian variations in the accuracy of visual working memory performance using a spatial working memory task. The variation of visual working memory performance showed a positive correlation with the rectal temperature by a 3-hour delay^25^. Moreover, the time of day effect can be modulated by participants’ chronotype. For example, the performance of cognitive tasks is better when individuals are tested at their preferred time (e.g. the morning for morning-type people, and the evening for evening-type people)^26^. Additionally, some other daily functions and activities, such as glucose absorption^27^, exposure to computer screen light^28^, a single session of physical exercise^29^ and so on, can also immediately influence cognitive performance. Therefore, it can be expected that time of day might also affect the estimation of visual working memory capacity.

In summary, some studies have examined the test-retest reliability of visual working memory capacity estimation^18,23^. However, the reliability of the lateralized whole-display-probe task has not yet been examined. Most electroencephalography (EEG) studies of visual working memory have used a “lateralized” variant of the whole-display-probe task, in which participants pay attention to stimuli in a hemi-field and ignore the distractors from the contra-lateral hemi-field. According to Pailian and Halberda’s work^20^, reliabilities can be different in different visual working memory tasks. Thus, the present study aimed to examine the test-retest reliability of visual working memory capacity estimation with a lateralized whole-display-probe change detection task. Additionally, the present study also examined whether variation in testing time is an important source of test-retest variation for visual working memory capacity.

## Method

### Participants

96 subjects participated in this study (18~28 years old; 48 females). Before the beginning of the experiment, they signed the informed consent form and received monetary compensation. All of the participants had normal or corrected to normal vision. This study was approved by the Institutional Review Board of the Institute of Psychology, Chinese Academy of Sciences. Moreover, all experiments were performed following relevant guidelines and regulations.

### Apparatus, stimuli and procedure

The present study adopted the change detection paradigm to measure visual working memory capacity^2^. The stimuli were presented on a 19 inch CRT display with a resolution of 1280 × 1024 pixels at a refresh rate of 75 Hz. Participants were seated 60 cm from the display with their heads rested on a chin-rest.

**Figure 1:**
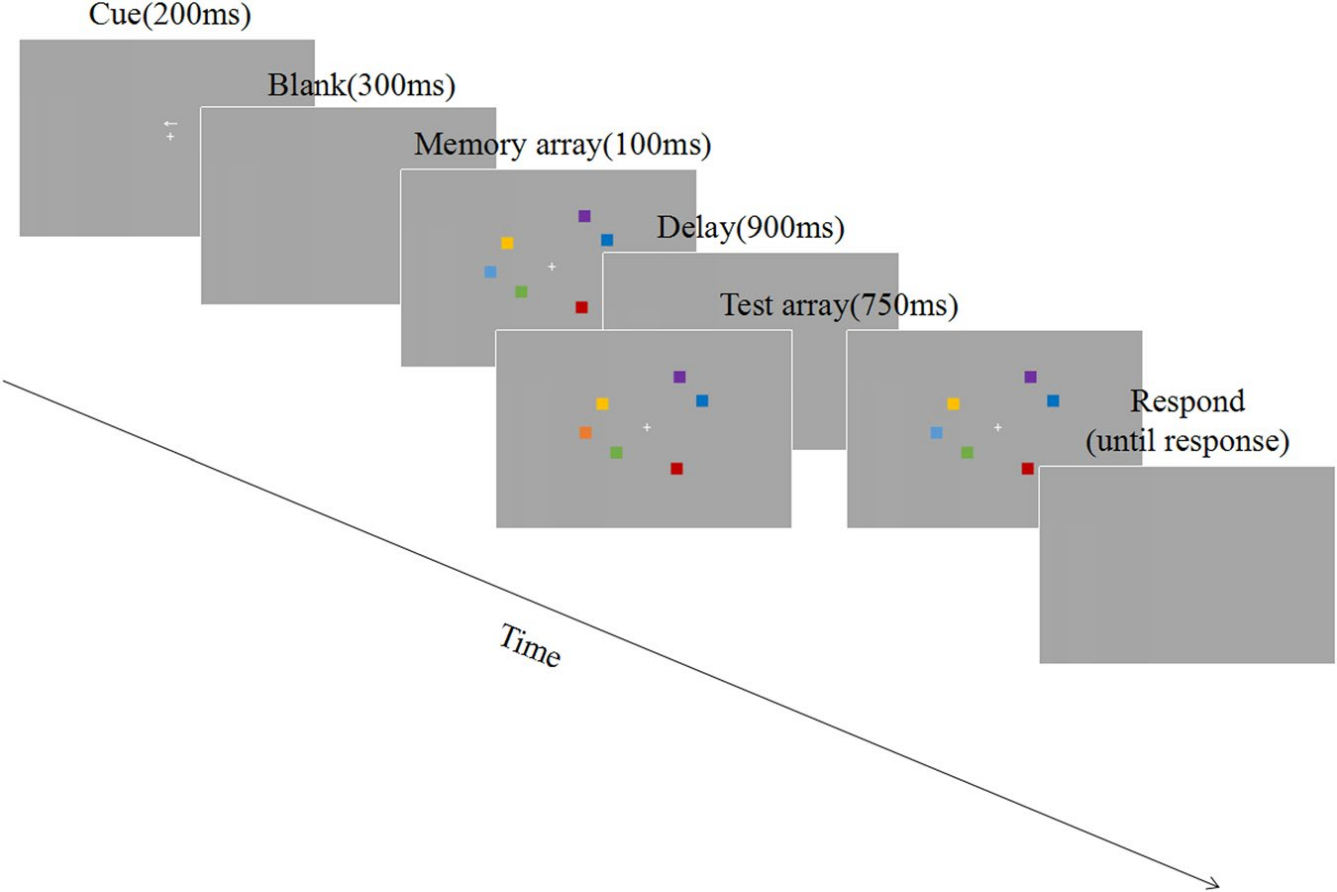
The schematic illustration of events in a trial.

The events of a trial are illustrated in Fig. 1. In each trial an arrow was presented above a central fixation point for 200 ms, pointing to either the left or right to indicate a cued hemi-field. After a 300 ms blank interval, the first array of 4, 6, 8, 10, or 12 colored squares appeared on the gray background for 100 ms. The size of each square was 0.65° * 0.65°, with a minimum distance of 2° between two squares. An equal number of colored squares were distributed in the left and right hemi-fields. Participants were instructed to only memorize the colored squares in the cued hemi-field. Then a blank screen appeared for 900 ms, which was followed by the second array of colored squares for 750 ms. The second array of squares was either identical to the first array or different from the first array in that one square in the cued hemi-field changed color. Then a blank screen remained until the participant responded by pressing corresponding keys. Since participants only had to memorize the squares in the cued hemi-field, the memory set size was 2, 3, 4, 5 or 6.

The first 35 participants were tested with memory set sizes of 2, 3, 4 or 5. However, the capacity estimate for two participants equaled 5, indicating that their working memory capacity might exceed the largest memory set size of 5. Thus, the largest memory set size was increased from 5 to 6. The other 61 subjects were tested with memory set sizes of 2, 3, 4, 5 or 6.

In the task, there were 100 trials for each set-size, and all trials were presented randomly. There was a 30-second break after every 100 trials. Each participant had to take the same test twice with a separation of 3 to 16 days.

### Working memory capacity measurement

According to Pashler^30^, the capacity of visual working memory is measured by the K value, the formula of which is as below. Compared to the other commonly used method proposed by Cowan^31^, Pashler’s method is more appropriate for the current paradigm^32^. The formula is as follows in equation 1:

1 $$K=setsiz{e}^{\ast }(P(hit)-P(FA))\div(1-P(FA))$$

where P(Hit) = hits/(hits + misses), and P(FA) = false alarms/(false alarms + correct rejections).

In addition to the K values of each set size, we also computed the average K value (K_mean_) and maximum K value (K_max_) across all set sizes as estimations for each participant’s visual working memory capacity.

To assess the test-retest reliability of K estimates, correlations between K estimates of the two tests were calculated. The test-retest reliability was considered good when r ≥ 0.71; fair 0.51 ≤ r ≤ 0.70; weak 0.31 ≤ r ≤ 0.50; little or none r ≤ 0.3^33^.

### Analysis of the time of day effect

The 96 subjects were divided equally into 2 groups based on the median time difference (48 minutes) between the two tests. The Same-time group (48 subjects, the time difference ranged from 0~44 minutes, mean = 13.23 min, sd = 10.02) and Different-time group (48 subjects, ranging from 52~688 minutes, mean = 217.44 min, sd = 162.95). The test-retest correlation coefficients of the two groups were compared by using Snedecor’s method^34^. This method compares correlations of two independent samples on the same pair of variables by using the following formula 2:

2 $$Z=\frac{{Z}_{1}-{Z}_{2}}{\frac{1}{({n}_{1}-3)+({n}_{2}-3)}}$$

Z_1_ and Z_2_ are Fisher’s Zs^35^ for correlation coefficients. Also, n_1_ and n_2_ are the sample sizes of the two independent samples.

## Results

### Descriptive statistics

The hit rates, false alarm rates, and K values under each memory set size for the two tests are listed in Table 1.

**Table 1:** The means and standard deviations of hit rates, false alarm rates, and K values.

		Set size2 N = 96	Set size3 N = 96	Set size4 N = 96	Set size5 N = 96	Set size6 N = 61
1^st^ test	P(Hit)	0.932 (0.064)	0.888 (0.102)	0.830 (0.125)	0.772 (0.166)	0.723 (0.157)
	P(FA)	0.044 (0.046)	0.069 (0.068)	0.116 (0.091)	0.136 (0.113)	0.148 (0.109)
	K	1.786 (0.211)	2.473 (0.443)	2.923 (0.705)	3.243 (1.154)	3.699 (1.225)
2^nd^ test	P(Hit)	0.952 (0.062)	0.923 (0.089)	0.907 (0.072)	0.882 (0.129)	0.839 (0.142)
	P(FA)	0.038 (0.102)	0.046 (0.096)	0.089 (0.114)	0.093 (0.112)	0.112 (0.098)
	K	1.865 (0.184)	2.665 (0.425)	3.344 (0.534)	4.046 (0.913)	4.744 (1.009)

### Test-retest improvement

**Figure 2:**
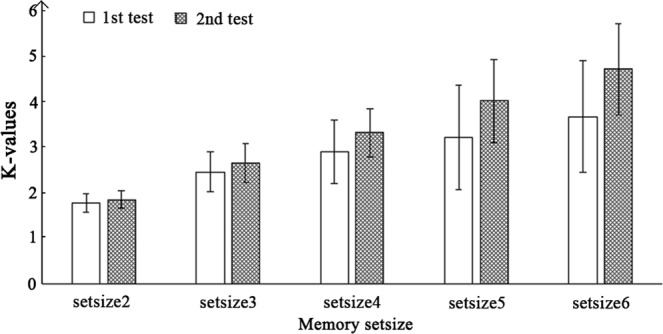
The average K values for each set size in the two tests.

The K values were submitted to a repeated-measures ANOVA with memory set size and test-retest as two within-subject variables (see Fig. 2). Results showed a significant main effect of test-retest, F = 103.104, p < 0.001, *η*^2^_*p*_ = 0.632, with the larger K in the second test than in the first test. The main effect of memory set size was also significant, F = 220.563 p < 0.001, *η*^2^_*p*_ = 0.935, with larger K values as set size increased. The interaction was also significant, F = 48.566, p < 0.001, *η*^2^_*p*_ = 0.647. Further analysis showed that test-retest improvements were significant at all levels of memory set size (all *p*s < 0.05).

The test-retest improvements of K values were possibly due to the enhancement of hit rate or the decline of the false-alarm rate. To address this, we undertook ANOVA analysis on both hit rate and false-alarm rate as for K values. Results showed that hit rate (F = 49.081, p < 0.001, *η*^2^_*p*_ = 0.454) was improved in the second test compared with the first test. Also, the false-alarm rate in the second test (F = 9.822, p = 0.003, *η*^2^_*p*_ = 0.141) was reduced compared with the first test.

### Test-retest reliability

Pearson product-moment correlations between the K values of the two tests under each set size are listed in Table 2. Results showed that at all set size levels, the K values in the second test were significantly correlated with the corresponding K values in the first test.

**Table 2:** Pearson’s correlations between Ks for each corresponding set size in the two tests.

	K_(T2-set size2)_	K_(T2-set size3)_	K_(T2-set size4)_	K_(T2-set size5)_	K_(T2-set size6)_	K_(T2-mean)_	K_(T2-max)_
K_(T1-set size2)_	0.505	0.399	0.386	0.363	0.421	0.493	0.432
K_(T1-set size3)_	0.506	0.502	0.472	0.497	0.494	0.590	0.526
K_(T1-set size4)_	0.332	0.363	0.572	0.543	0.544	0.564	0.536
K_(T1-set size5)_	0.354	0.339	0.449	0.647	0.676	0.592	0.614
K_(T1-set size6)_	0.393	0.392	0.498	0.618	0.757	0.727	0.768
K_(T1-mean)_	0.436	0.438	0.546	0.633	0.730	0.704	0.705
K_(T1-max)_	0.400	0.393	0.524	0.625	0.757	0.691	0.715

T1-set size 2 = memory set size 2 in the first test. Similarly, T2-set size3 = memory set size 3 in the second test.

All correlations are significant at p ≤ 0.01.

**Figure 3:**
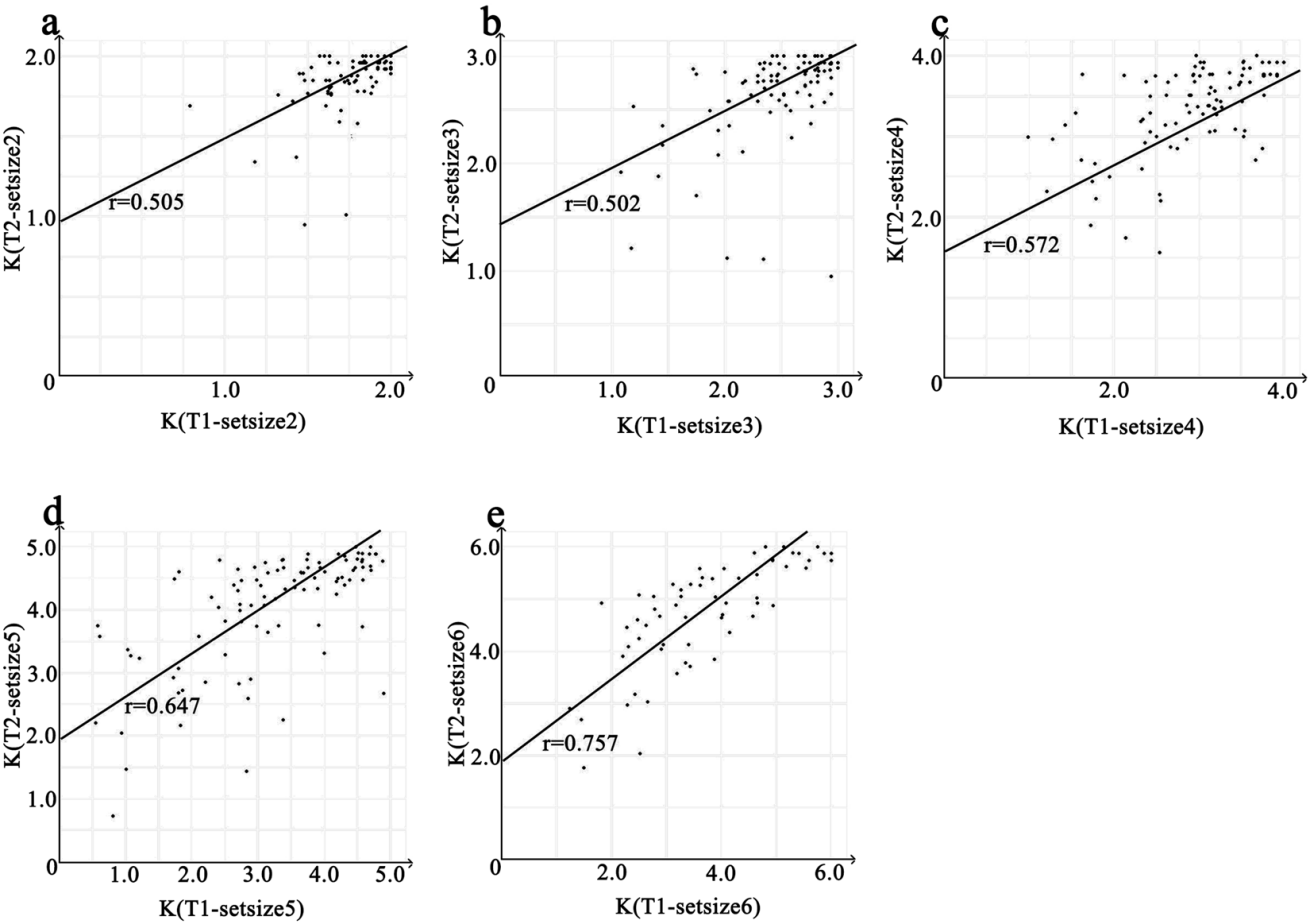
The test-retest correlations between Ks for each set size in the two tests. Panel a illustrates the correlations between K for set size 2 in the two tests. Panel b illustrates the correlations between K for set size 3 in the two tests. Panel c illustrates correlations between K for set size 4 in the two tests. Panel d illustrates correlations between K for set size 5 in the two tests. Panel e illustrates correlations between K for set size 6 in the two tests.

Also, as the memory set size increased, the test-retest reliability coefficients for each corresponding set size also showed a rising trend (Fig. 3). To examine whether this trend was significant, the modified Pearson-Filontest^36^ as in equations (3) and (4), was used to compare test-retest reliability coefficients across set sizes. This test is to compare correlation coefficients within the same sample but with non-overlapping pairs of variables^37^.

3 $${Z}_{PF}=\sqrt{\frac{n-3}{2}}\times \frac{{Z}_{AB}-{Z}_{XY}}{\sqrt{1-\frac{kPF}{2(1-{r}_{AB}^{2})(1-{r}_{XY}^{2})}}}$$

where *kPF* is computed by

4 $$\begin{array}{rcl}kPF & = & ({r}_{AX}-{r}_{BX}\times {r}_{AB})\times ({r}_{BY}-{r}_{BX}\times {r}_{XY})+({r}_{AY}-{r}_{AX}\times {r}_{XY})\\  &  & \times \,({r}_{BX}-{r}_{AX}\times {r}_{AB})+({r}_{AX}-{r}_{AY}\times {r}_{XY})\times ({r}_{BY}-{r}_{AY}\times {r}_{AB})\\  &  & +\,({r}_{AY}-{r}_{AB}\times {r}_{BY})\times ({r}_{BX}-{r}_{BY}\times {r}_{XY})\end{array}$$

*A, B, X, Y* represent the two non-overlapping pairs of variables. *Z*_*AB*_ and *Z*_*XY*_ are the Fisher-z transformed result of *r*_*AB*_ and *r*_*XY*_.

Results showed that only the test-retest reliability coefficients for set size 6 were significantly higher than those for set size 2 (Tables 3 and 4). The sample size was 61 in each pair comparison including set size 6, and 96 in those not including set size 6.

**Table 3:** Comparisons between test-retest reliability coefficients for each set size.

	r_set size3_ = 0.502	r_set size4_ = 0.572	r_set size5_ = 0.647
r_set size2_ = 0.505	Z = 0.029	Z = −0.064	Z = −1.488
	p = 0.976	p = 0.506	p = 0.137
	N = 96	N = 96	N = 96
r_set size3_ = 0.502		Z = −0.708	Z = −1.541
		p = 0.479	p = 0.123
		N = 96	N = 96
r_set size4_ = 0.572			Z = −0.851
			p = 0.395
			N = 96

**Table 4:** Comparisons between test-retest reliability coefficients for each set size.

	r_set size2_ = 0.512	r_set size3_ = 0.670	r_set size4_ = 0.614	r_set size5_ = 0.708
r_set size6_ = 0.757	Z = −2.307	Z = −0.966	Z = −1.489	Z = −0.577
	p = 0.021	p = 0.334	p = 0.137	p = 0.564
	N = 61	N = 61	N = 61	N = 61

### The internal consistency across memory set sizes within a single test

The within-test consistency across set sizes refers to the correlations between K values for different memory set sizes within a single test. The correlations between K values for different memory set sizes in the first test are listed in Table 5. All of the correlations were medium or high.

**Table 5:** Pearson’s correlations between Ks for each set size in the first test.

	K_(T1-set size3)_	K_(T1-set size4)_	K_(T1-set size5)_	K_(T1-set size6)_
K_(T1-set size2)_	0.710	0.562	0.526	0.559
K_(T1-set size3)_		0.744	0.728	0.618
K_(T1-set size4)_			0.803	0.696
K_(T1-set size5)_				0.793

All correlations are significant at *p* ≤ 0.005.

We also examined the within-test consistency across set sizes in the second test. The correlations between K values for different memory set sizes in the second test are listed in Table 6. All of the correlations were medium or high.

**Table 6:** Pearson’s correlations between Ks for each set size in the second test.

	K_(T2-set size3)_	K_(T2-set size4)_	K_(T2-set size5)_	K_(T2-set size6)_
K_(T2-set size2)_	0.722	0.498	0.448	0.427
K_(T2-set size3)_		0.593	0.551	0.431
K_(T2-set size4)_			0.710	0.512
K_(T2-set size5)_				0.780
K_(T2-set size6)_				

All correlations are significant at p ≤ 0.01.

### The time of day effect on test-retest reliability

The test-retest reliability coefficients for the two groups (the same time of day v.s. different time of day) are listed in Table 7. We examined whether participants who took the two tests at the same time of day showed a higher test-retest reliability than participants who undertook the two tests at different times of day. As Table 7 shows, in the K(setsize3), K(setsize5), K(mean) and K(max) conditions, the test-retest reliability coefficients for the same time of day group were significantly higher than those for the different time of day group.

**Table 7:** Test-retest reliabilities for the same-time group and the different-time group.

		K_(setsize2)_	K_(setsize3)_	K_(setsize4)_	K_(setsize5)_	K_(setsize6)_	K_(mean)_	K_(max)_
Same-time of day	r	0.654	0.749	0.590	0.795	0.814	0.842	0.847
	N	48	48	48	48	37	48	48
Different-time of day	r	0.467	0.283	0.598	0.534	0.640	0.602	0.581
	N	48	48	48	48	24	48	48
Comparison between two groups	z	1.390	3.224	−0.059	2.320	1.371	2.522	2.758
	p	0.190	0.001	0.953	0.020	0.170	0.012	0.006

## Discussion

The present study examined the test-retest reliability of VWM capacity estimation by using the change detection paradigm. The test-retest correlations between K values for corresponding set sizes in the two tests were comparable to those in the verbal recall tasks, as found by Gathercole, *et al*.^13^, varying from fair to good. The results also showed a trend that the test-retest reliability increased as the set size became larger. In addition, we found that the test-retest reliability of VWM capacity estimation was higher when the two tests occurred at the same time of day than at different times of day.

The test-retest reliability of VWM capacity estimation (K values) was reasonably good in the present study, indicating that K values can be a relatively reliable index of individual VWM capacity. Secondly, a trend was found showing that the larger the memory set size, the higher the corresponding test-retest reliability for the K values. This trend was possibly due to a ceiling effect at set sizes 2 and 3 (see Fig. 2a for set size 2 and Fig. 2b for set size 3), which were lower than many participants’ true VWM capacity^4^. This ceiling effect made it difficult to detect individual differences among participants. Thus, it is important for future work to use a memory set size that is at least larger than 4 when estimating individual VWM capacity.

In this study, the performance of the second test was better than that of the first one, indicating a practice effect with a 3~16 day interval (the time between 2 tests) between the two tests. This finding is inconsistent with Johnson *et al.*’s^18^ research, which found no practice effect for visual working memory tests with a 1.5-year interval. It is possible that the practice effect waned with the greater interval between the two tests. However, the critical point of tim eat which practice effects disappear has yet to be studied. Thus, it is important for future work to allow for sufficient practice before formal measurement because the K value based on a short version of the change detection task might result in underestimation of VWM capacity. This conclusion is similar to that from Xu and colleagues’ work, which found that adjacent-session correlation increased along with session number within 31 sessions^23^. However, more factors able to influence retest reliability should be discussed. It is not practical to test research subjects dozens of times in every experiment.

Reasonably good correlations were found between different set sizes in the same test, in contrast to the quite low correlations between different set sizes in a previous study^20^. This discrepancy might be due to methodological differences between the studies. First, the included set sizes were different. The current research used memory set sizes of 2, 3, 4, 5, and 6, while Pailian and Halberda^20^ tested memory set sizes of 2, 4 and 8. The larger step size of the memory set sizes in the Pailian and Halberda^20^ study might account for the lower correlations between different set sizes. Second, a larger sample was included in the current research (96 participants) compared with the 14 participants in Pailian and Halberda’s^20^ study.

Test-retest reliability was higher when the two tests occurred at the same time of day than at different times of day. This time of day effect was significant for the mean and maximum values of Ks across set sizes, and also for the values of K for set sizes 3 and 5. According to a meta-analysis in Schmidt and colleagues’ review, cognitive performance in a phonological working memory task^25^, visual working memory task^25^ and visual selective attention^38^ can be influenced by circadian rhythm^26^. Also cognitive performance can be affected by daily activities^27–29^. Therefore, at different times of day, participants’ performance varied in different directions, causing a reduced correlation of the test results, while tests taken at the same time of day had higher retest reliability. However, it is worth noting that the time of day effect on test-retest reliability was not significant for set sizes of 2, 4 and 6. There are several possible explanations for this variation. First, the K value for a specific set size might not be sufficiently reliable, especially for small set sizes. Second, time of day was a rough correlate of circadian rhythms and daily activities, which might also add variation to the results. Finally, the relatively smaller sample in set size 6 might explain the absence of a time of day effect. Thus, the time of day effect needs further examination.

In conclusion, with appropriate control, the estimation of VWM capacity based on the change detection task is highly reliable. The average or maximum K values across multiple set sizes are also reliable. Results from the current study also indicate that future research on measuring individual working memory capacity should use set sizes larger than 4, and should allow for sufficient practice before formal measurement.
